# A comparison of outcome measures used to report clubfoot treatment with the Ponseti method: results from a cohort in Harare, Zimbabwe

**DOI:** 10.1186/s12891-018-2365-3

**Published:** 2018-12-22

**Authors:** Tracey Smythe, Maxman Gova, Rumbidzai Muzarurwi, Allen Foster, Christopher Lavy

**Affiliations:** 1 International Centre for Evidence in Disability, London School of Hygiene & Tropical Medicine, Keppel Street, London, WC1E7HT UK; 2 Department of Surgery, Parirenyatwa Group of Hospitals, Harare, Zimbabwe; 3 Rehabilitation Department, Parirenyatwa Group of Hospitals, Harare, Zimbabwe; 4 Nuffield Department of Orthopaedics Rheumatology and Musculoskeletal Science, University of Oxford, Oxford, UK

**Keywords:** Clubfoot, CTEV, Measurement, Quality, Ponseti, Evaluate, Indicator, Low resource, Tool

## Abstract

**Background:**

There are various established scoring systems to assess the outcome of clubfoot treatment after correction with the Ponseti method. We used five measures to compare the results in a cohort of children followed up for between 3.5 to 5 years.

**Methods:**

In January 2017 two experienced physiotherapists assessed children who had started treatment between 2011 and 2013 in one clinic in Harare, Zimbabwe. The length of time in treatment was documented. The Roye score, Bangla clubfoot assessment tool, the Assessing Clubfoot Treatment (ACT) tool, proportion of relapsed and of plantigrade feet were used to assess the outcome of treatment in the cohort. Inter-observer variation was calculated for the two physiotherapists. A comparative analysis of the entire cohort, the children who had completed casting and the children who completed more than two years of bracing was undertaken. Diagnostic accuracy was calculated for the five measures and compared to full clinical assessment (gold standard) and whether referral for further intervention was required for re-casting or surgical review.

**Results:**

31% (68/218) of the cohort attended for examination and were assessed. Of the children who were assessed, 24 (35%) had attended clinic reviews for 4–5 years, and 30 (44%) for less than 2 years. There was good inter-observer agreement between the two expert physiotherapists on all assessment tools. Overall success of treatment varied between 56 and 93% using the different outcome measures. The relapse assessment had the highest unnecessary referrals (19.1%), and the Roye score the highest proportion of missed referrals (22.7%). The ACT and Bangla score missed the fewest number of referrals (7.4%). The Bangla score demonstrated 79.2% (95%CI: 57.8–92.9%) sensitivity and 79.5% (95%CI: 64.7–90.2%) specificity and the ACT score had 79.2% (95%CI: 57.8–92.9%) sensitivity and 100% (95%CI: 92–100%) specificity in predicting the need for referral.

**Conclusion:**

At three to five years of follow up, the Ponseti method has a good success rate that improves if the child has completed casting and at least two years of bracing. The ACT score demonstrates good diagnostic accuracy for the need for referral for further intervention (specialist opinion or further casting). All tools demonstrated good reliability.

**Electronic supplementary material:**

The online version of this article (10.1186/s12891-018-2365-3) contains supplementary material, which is available to authorized users.

## Background

Clubfoot, or congenital talipes equinovarus, is a condition that is present at birth in which the foot is in a rigid turned-in position. Corrective treatment of a high quality remains a key requirement for reducing disability and improving function related to the deformity. Over the past decades there has been an increase in the use of the Ponseti method to correct clubfoot [1]. This method involves the simultaneous correction of three components of the clubfoot deformity through manipulation and serial casting. The equinus (downward pointing of the foot) is corrected last, often with a percutaneous achilles tenotomy. This is followed by long term use of a foot abduction brace at night to maintain the foot position [2]. Despite the global trend toward increased use of the Ponseti method, there remains variation in how success of clubfoot treatment is measured [3, 4].

The Ponseti method is administered by locally trained therapists in resource constrained settings in Africa [5]. These clubfoot therapists often work alone and have no specialised physiotherapy or surgical support present in the clinics or nearby. It is important that they have a user friendly assessment system with agreed criteria for when treatment is not working and referral to a specialist for further management is indicated.

No globally accepted outcome scoring system exists to inform locally trained clubfoot therapists of the need for referral for further intervention. The most frequently used approach to measuring whether the Ponseti method has been successful (or not) is clinical assessment. In sub-Saharan Africa 68 to 98% of cases are reported to have a successful outcome with the Ponseti method [4]. This study aims to compare the results of the Ponseti method of clubfoot management at three to five years from initial correction using five different outcome measures. We explore the diagnostic accuracy of the outcome measures, which is the ability of the assessments to discriminate between the need for referral for further intervention and a successful outcome [6]. For methodology review, outcome score results in this study are compared with a reference standard of ‘true’ treatment success status (defined by full clinical assessment). The results are categorised as true positive, false positive (referred but not needed), true negative, and false negative (should have been referred but was missed) [7]. Sensitivity of the scoring system relates to the proportion of the children who need referral for further intervention and who are correctly classified by the outcome measure as requiring referral. Specificity is the proportion of children who do not need referral and who are correctly classified as not requiring referral by the outcome measure. Positive predictive value and negative predictive value are useful to understand the probability that a child with a given positive or negative outcome score result has the need for referral for further intervention and are therefore correctly classified.

## Methods

### Study design and population

This study was conducted and reported according to established STARD (Standards for Reporting of Diagnostic Accuracy Studies) guidelines [8] (Additional file 1). A cohort study of 218 children with idiopathic clubfoot was conducted in 2016. The children were managed with manipulation and casting at Parirenyatwa Hospital, Harare and the results are published elsewhere [9]. All children with a diagnosis of unilateral or bilateral idiopathic clubfoot who started treatment with the Ponseti method at the study hospital between 22nd March 2011 and 23rd April 2013 (25 months) were included in the cohort. The only exclusion criterion was foot conditions other than idiopathic clubfoot, for example clubfoot associated with neural-tube defects such as spina-bifida.

### Sampling technique

The phone numbers of all carers of the cohort children were extracted from the clinic records in January 2017 and contact with them was attempted at least three times. Caregivers and their children were invited to attend the study. The children were between 3.5 and 5 years from initial casting.

### Ethics, consent and permissions

Ethical approval for this study was granted by the Medical Research Council of Zimbabwe (MRCZ) and the London School of Hygiene & Tropical Medicine (LSHTM) (ref:11132 /RR/4725). All children and their caregivers were read an information sheet about the study and given an opportunity to ask questions. If they agreed to participate, written consent was taken from the caregiver who remained present throughout the assessment as per national requirements. Transport costs were reimbursed and referral services available in Harare were mapped pre-emptively to ensure appropriate onward referral for any children that required further intervention.

### Data collection

Two physiotherapists who are experienced in co-ordinating national clubfoot programmes reviewed the assessment tools over three days for contextual relevance. The questionnaires were available in English and Shona and were cognitively tested. We used five outcome methods, three that give a score, and two that give a binary (success/failure) outcome. The Roye score [10] is a self-reported measurement that is used in high income settings. The Bangla clubfoot assessment tool [11] and the Assessing Clubfoot Treatment (ACT) score [12] combine physical assessment and parent reported outcome measures, and have been developed for low resource settings. The Bangla score includes a functional assessment. The two binary outcomes were assessment of a plantigrade foot [5] and the relapse pattern [13]. The study protocol was pilot tested for suitability in July 2016. Children were examined independently in January 2017 by the two physiotherapists and a decision was made if referral for further intervention (re-casting or surgical review) was required. Clinical examination composed observation, physical assessment and functional review; it included assessment of passive and active range of motion (plantiflexion, dorsiflexion, eversion, inversion of the foot, and knee extension), muscle strength tests of the calf and evertors of the foot, heel raises, squatting ability and gait analysis (walking and running).

### Data management and analysis strategy

The data were entered into a Microsoft Excel 2000 (Microsoft Inc., Redmond, Washington) software package. Data were analysed using Stata 14.1 (Stata-Corp 4905, Lakeway Drive College Station, Texas 77, 845, USA). Statistical significance was set at the 95% confidence level. The inter-observer variation for the measurement of the physical assessment tools was assessed i.e. Intra-class correlation coefficient (ICC) ≥0.75 [10]. Outcomes of children who had completed casting and ≥ two years of bracing were compared to all of the children who were followed up, and to those who had only completed casting. A two-tailed paired t-test was used to assess the mean difference between the outcome measures of Roye, Bangla and ACT scores. Fisher’s exact test of independence was used to assess the difference in proportion of children with an outcome of relapse and plantigrade foot. The five measures were compared against the standard of whether referral for further intervention was required (for re-casting or surgical review) as defined by a consensus agreement of two expert physiotherapists with experience of managing clubfoot in countries in Africa. Sensitivity, specificity, positive and negative predictive values were calculated for the five measures and compared to full clinical assessment (gold standard). The threshold for diagnostic accuracy was based on previous studies and was defined prior to the study. It was set at 70% for the three scores with continuous scales [14] and positive/negative for the binary outcomes [7].

## Results

**Fig. 1:**
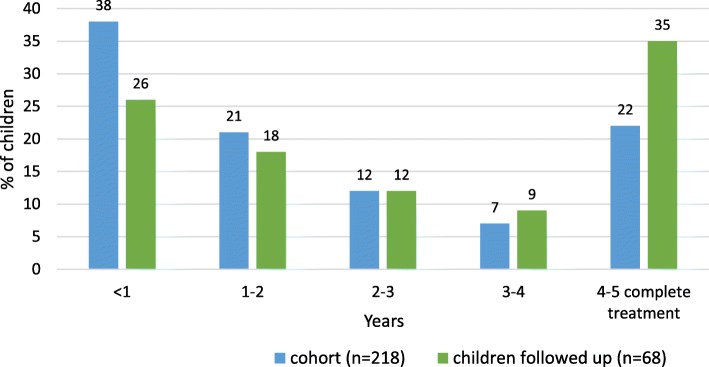
Length of time child attended clubfoot clinic appointments

31% (68/218) of the cohort attended for review and were assessed. 50 (73%) children were boys and 18 (27%) were girls. There were 35 (51%) bilateral and 33 (49%) unilateral clubfeet. Tenotomies had been performed in 52 (76%) cases and the average number of casts to correction was 6.9 (5.9–8.0 casts). The average length of time attending appointments from initial review was 30 months (26 – 35 months). Of the children followed up, 24 (35%) attended clinic reviews for 4–5 years (Fig. 1).

All tools demonstrated good reliability, with an intra-class coefficient (ICC) of ≥0.82 on all criteria (Table 1). An ICC of 1.00 demonstrates perfect correlation.

**Table 1:** Inter-observer variation for outcome measures

Outcome Measure	ICC	95%CI
Bangla Score		
1. Happy with child’s feet?	0.96	0.94–0.98
2. Recommmend to others?	1.00	1.00
3. Does child play with others?	1.00	1.00
4. Does child wear shoes of choice?	0.97	0.95–0.98
5. Does child have pain?	1.00	1.00
6. Squatting	1.00	1.00
7. Walking	1.00	1.00
8. Running	1.00	1.00
9. Up/down stairs	1.00	1.00
10a. Heel position L	0.94	0.88–0.97
10b. Heel position R	0.98	0.97–0.99
11a. Ankle range L	0.82	0.66–0.90
11b. Ankle range R	0.99	0.98–0.99
Relapse assessment		
1A - reduced DF	0.96	0.93–0.98
2A - fixed equinus	1.00	1.00
1B - dynamic supination, flex add	0.88	0.79–0.93
2B - fixed forefoot add	1.00	1.00
3–2 or more deformities	1.00	1.00
ACT score		
1. Foot is plantigrade	0.99	0.98–0.99
2. Does child complain of pain?	1.00	1.00
3. Can child wear shoes of choice?	0.99	0.99–1.00
4. How satisfied is the carer?	1.00	1.00
Plantigrade foot	0.99	0.98–0.99
Roye score	1.00	1.00

(ICC > 75 = good consistency)

**Table 2:** Results of cohort of children followed up (n = 68)

Outcome measure	Poor < 49 *N* (%)	Fair: 50–69 *N* (%)	Good: 70–84 *N* (%)	V Good: 85–100 *N* (%)
Roye^a^	4 (6%)	3 (5%)	20 (30%)	39 (59%)
Total Roye^a^	7 (11%)		59 (89%)	
Bangla	12 (17%)	16 (24%)	15 (22%)	25 (37%)
Total Bangla	28 (41%)		40 (59%)	
ACT score	7 (10%)	12 (18%)	13 (19%)	36 (53%)
Total ACT score	19 (28%)		49 (72%)	
	Cannot achieve plantigrade		Achieved plantigrade or better	
Plantigrade	13 (19%)		55 (81%)	
Any form of relapse	Yes		No	
	30 (44%)		38 (56%)	
Requires referral for further intervention	Yes		No	
	16 (24%)		52 (76%)	

^a^data missing for 2 children

In the children who were followed up (*n* = 68) the success of treatment with different scores varied between 56 and 89% (Table 2). In the children who completed casting (*n* = 63) it was between 57 and 93%; and in the children who completed casting and at least two years of bracing (*n* = 38) it was from 58 to 97% (Table 3). The individual category calculations for each outcome measurement are in Additional files 2, 3, 4 and 5.

**Table 3:** Results of cohort of children followed up who completed > 2 years bracing (n = 38)

Outcome measure	Poor < 49 *N* (%)	Fair: 50–69 *N* (%)	Good: 70–84 *N* (%)	V Good:85–100 *N* (%)
Roye^a^	1 (3%)	1 (3%)	10 (28%)	24 (66%)
Total Roye^a^	2 (6%)		34 (94%)	
Bangla	3 (8%)	10 (26%)	9 (24%)	16 (42%)
Total Bangla	13 (34%)		25 (66%)	
ACT score	1 (3%)	5 (13%)	9 (24%)	23 (60%)
Total ACT score	6 (16%)		32 (84%)	
	cannot achieve plantigrade		Achieved plantigrade or better	
Plantigrade	1 (3%)		37 (97%)	
Any form of relapse	Yes		No	
	16 (42%)		22 (58%)	
Requires referral for further intervention	Yes		No	
	5 (13%)		33 (87%)	

^a^data missing from 2 children

**Fig. 2:**
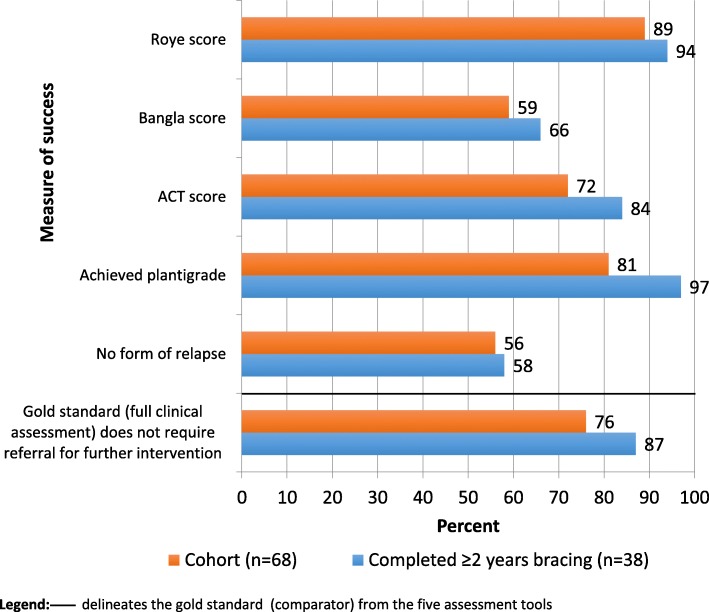
Comparison of outomes to measure success against full clinical assessment

The proportion of children with relapse and the Bangla tool had the lowest good outcome results of 56 and 59% respectively. Figure 2 demonstrates the variation in outcome when compared to full clinical assessment (the gold standard illustrated in the first row of the figure). 87% (33/38) children who completed ≥2 years bracing were assessed as successfully treated with full clinical assessment. The scores that demonstrate a higher success (Plantigrade: 97% and Roye score: 94%) miss cases that require further intervention. The scores that demonstrate a lower success (Relapse: 58% and Bangla: 66%) are restrictive in the measurement of success.

There was strong evidence for a difference between the outcomes of the Roye score and the Bangla score (*p* < 0.0001), the Roye and the ACT score (*p* = 0.0013), and the ACT and Bangla score (p < 0.0001). It follows that none of these assessments can provide essentially the same estimate of success as the other measures.

There was a difference in the relative proportion of the cohort with relapse and plantigrade foot when assessed with Fischer’s exact test (*p* = 0.012). The binary outcomes are therefore not interchangeable.

No adverse events occurred as a result of any of the outcome measures undertaken. When compared to the standard of full clinical assessment and the subsequent decision on the need for referral for further intervention, the Roye score had a sensitivity of 31.8% (95%CI: 13.9–54.9%) and a specificity of 100% (95%CI: 92–100%), with positive and negative predictive values of 100 and 74.6% respectively. The Bangla score demonstrated 79.2% (95%CI: 57.8–92.9%) sensitivity and 79.5% (95%CI: 64.7–90.2%) specificity with 67.9% positive predictive and 87.5% negative predictive values, and the ACT score had 79.2% (95%CI: 57.8–92.9%) sensitivity and 100% (95%CI: 92–100%) specificity in predicting the need for referral, with positive and negative predictive values of 100 and 89.8% respectively. Of the 44 children that did not require referral for further intervention, all achieved plantigrade or more (positive predictive value: 100%) and of those who did require referral (*n* = 24), 14 were identified with the plantigrade assessment (achieved less than plantigrade). The relapse score was most restrictive in identifying good outcome. False positive and false negative scores are displayed in Table 4.

**Table 4:** A comparison of measurement methods with the need for referral for further intervention

Method	Unnecessary referral (false positive) *n* (%)	Missed Referral (false negative) *n* (%)
Roye (*n* = 66)	0 (0%)	15 (22.7%)
Bangla (*n* = 68)	10 (14.7%)	5 (7.4%)
ACT (*n* = 68)	1 (1.5%)	5 (7.4%)
Plantigrade (*n* = 68)	0 (0%)	10 (14.7)
Relapse (*n* = 68)	13 (19.1%)	6 (8.8%)

## Discussion

This study found that five scoring systems that are used to report outcomes of clubfoot treatment provided a wide spectrum of success (from 56 to 89% of cases) in a cohort with 3.5–5 years of follow up. When compared with the standard of clinical assessment, missed referrals ranged from 7.4% (the Bangla and ACT scores) to 22.7% (the Roye score). The measurements assess different aspects of clubfoot correction, from parent reported outcome measures (the Roye score) to scores that include physical assessment (the Bangla and ACT score) and single measurements (plantigrade foot and evidence of recurrence). Success improves in all measures with the completion of casting and at least two years of bracing.

### Comparison to previous studies

There are limited studies that compare measurement tools in the same patient against which to compare our findings. However, success of treatment in this cohort is similar to other studies in sub-Saharan Africa (between 63 and 98% of cases) [9]. Non-adherence and surgical intervention, often defined as failure, are reported to vary from 7 to 61% and 3–39.4% [15] respectively. Ponseti and Laaveg [16] describe a scoring system that rates functional results as satisfactory in 88.5% of feet. Further studies describe success using the Ponseti and Laaveg system as 89.3% [17]. The criteria includes the need for a goniometer and the tool was therefore not included in evaluation of this cohort.

### Use of outcome measures

The ease of use and rate of incorrect classification in the tools used to measure success need to be considered when selecting an outcome measure. Single item scales for assessment of individual children require no further calculation and may be easier to use in clinics (such as plantigrade foot or evidence of relapse), however their simplicity may not allow a full assessment of success. Multi-scale items prove difficult to transform into useful statistics without technology and are unlikely to be routinely used in clinics. This study found no clear agreement between the different outcome measurements in use.

All of the assessments used in this study have limitations. The Roye score has been validated in high income settings and parents in our study reported difficulty in answering the question of “How often does your child have problems finding shoes that he or she likes?” as it was understood to be related to the availability of a variety of shoes. The Bangla score took the longest time to transform with statistical analysis. Acceptability and feasibility of the ACT score is needed to be studied in future research. The ACT score is likely easy to teach, however this is unknown as the examiners were physiotherapists; the time taken for other cadres of health workers to use the ACT tool is also unknown. With regard to the relapse score, Bhaskar et al. (2013) considered ankle dorsiflexion < 15 degrees with knee in extension as grade IA relapse. This may be a reason for the restriction in defining good outcome as an evaluation of 85 normal feet in children found that the mean ankle dorsiflexion was 12.8 degrees with knees in extension [18]. Greater than 15 degrees may therefore be difficult to achieve.

### Relationship between the outcome measures and clinical assessment

The Bangla and ACT tool were most helpful in predicting the need for referral for further intervention (specialist opinion or for further manipulation and casting). The five referrals that were missed with the ACT score were children who required review of a mobile curvature of the lateral border of the foot or supination in swing phase, neither of which are assessed with the score. Despite this, the ACT tool demonstrates the best diagnostic accuracy for the need for referral for further intervention.

### Strengths and limitations of study

This study reports on five measurements of success in a cohort at 3.5–5 years from initial treatment. Repeat phone calls facilitated assessments when caregivers were initially unavailable. Two independent raters reduced the likelihood of reporting bias and all outcome measures were verified by the reference standard. The threshold for diagnostic accuracy was based on previous studies and was defined prior to the study. There were also study limitations. No distinction between a clubfoot that may not have been fully corrected and a relapsed clubfoot was made, and all cases with elements of the deformity were classified with the relapse score, which may be a source of potential bias that underestimates the accuracy of the relapse score. The tools were chosen based on ease of use in low resource high volume clinics and were not all initially developed to identify need for referral for further intervention.

### Implications for practice

Task shifting and task sharing between orthopaedic and non-specialised health workers in some clinics means that outcome measures are even more important as teams expand. As older children are being treated with the principles of the Ponseti method [19], expert guidance on assessment and measurement in these cases is needed. The Roye score is overly optimistic of good outcomes, the Bangla score is restrictive in identifying good outcome, and the ACT score most closely aligns to clinical examination. However, the Bangla, relapse and ACT scores closely agree on false negatives and have the least chance of missing recurrence; the Bangla score and the relapse score over-estimate referral needs compared to the ACT score.

## Conclusions

In this small comparative study, missed referrals ranged from 7.4% (the Bangla and ACT scores) to 22.7% (the Roye score) when compared with the standard of clinical assessment. Ease of use and the cost of false positives need to be considered in the selection of a tool. All scores demonstrated good reliability. The Roye score will miss cases and the Bangla and the Relapse tools are restrictive in assessment of successful outcome. We found no clear agreement between the different scores in use. When compared to the normal practice of full clinical assessment, the measurement tool with the best evidence for diagnostic accuracy was the ACT tool.

## Additional files

Additional file 1: The STARD 2015 list. Standards for Reporting of Diagnostic Accuracy Studies guidelines. (DOCX 19 kb)

Additional file 2: Summary of outcomes: Roye score. Individual category calculations for the Roye score. (DOCX 24 kb)

Additional file 3: Summary of outcomes: Bangla score. Individual category calculations for the Bangla score. (DOCX 23 kb)

Additional file 4: Summary of outcomes: ACT score. .Individual category calculations for the ACT score. (DOCX 16 kb)

Additional file 5: Summary of outcomes: Relapse score. Individual category calculations for the Relapse score. (DOCX 15 kb)

## Abbreviations

ACT: Assessing Clubfoot Treatment
ICC: Intra-class correlation coefficient
