# Supplementary material for: A comparison of outcome measures used to report clubfoot treatment with the Ponseti method: results from a cohort in Harare, Zimbabwe
Source: BMC Musculoskelet Disord. 2018 Dec 22;19:450. doi: 10.1186/s12891-018-2365-3 (PMC6303847; doi:10.1186/s12891-018-2365-3)
Supplement: Supplementary file 1 — The STARD 2015 list. Standards for Reporting of Diagnostic Accuracy Studies guidelines. (DOCX 19 kb) [file 12891_2018_2365_MOESM1_ESM.docx]

**Additional File 1: The STARD 2015 list**

| **Section and topic** | **No** | **Item** |
| --- | --- | --- |
| **Title or abstract** | | |
|  | 1 | Identification as a study of diagnostic accuracy using at least one measure of accuracy (such as sensitivity, specificity, predictive values or AUC)  **A comparison of outcome measures used to report clubfoot treatment with the Ponseti method: the predictive ability of need for further intervention** |
| **Abstract** | | |
|  | 2 | Structured summary of study design, methods, results and conclusions |
| **Introduction** | | |
|  | 3 | Scientific and clinical background, including the intended use and clinical role of the index test  **In low resource settings through Africa the Ponseti method is administered by locally trained therapists. These therapists often work alone and have no specialised physiotherapy or surgical support present in the clinics or nearby. It is important that they have a user friendly assessment system with agreed criteria for when treatment is not working and referral to a specialist for further management is indicated.** |
|  | 4 | Study objectives and hypotheses  **We aim to:**   1. **compare the results of the Ponseti method of clubfoot management at three to five years from initial correction using five different outcome measures.** 2. **explore the diagnostic accuracy of the outcome measures (the ability of the assessments to discriminate between the need for referral for further intervention and a successful outcome)** |
| **Methods** | | |
| Study design | 5 | Whether data collection was planned before the index test and reference standard were performed (prospective study) or after (retrospective study)  **Data were collected before the index test and reference standard were performed** |
| Participants | 6 | Eligibility criteria  **Cohort study: there was one set of eligibility criteria for all study participants** |
|  | 7 | On what basis potentially eligible participants were identified (such as symptoms, results from previous tests, inclusion in registry)  **All children with a diagnosis of unilateral or bilateral idiopathic clubfoot who started treatment with the Ponseti method at the study hospital between 22nd March 2011 and 23rd April 2013 (25 months) were included in the cohort.** |
|  | 8 | Where and when potentially eligible participants were identified (setting, location and dates)  **Treated at Parirenyatwa Hospital, Harare between 22nd March 2011 and 23rd April 2013** |
|  | 9 | Whether participants formed a consecutive, random or convenience series  **The included study participants are a consecutive series of all patients evaluated for eligibility at the study location and satisfying the inclusion criteria** |
| Test methods | 10a | Index test, in sufficient detail to allow replication  **The index tests were performed as per guidelines in the published papers and references provided** |
|  | 10b | Reference standard, in sufficient detail to allow replication  **Children were examined independently in January 2017 by the two physiotherapists and a decision was made if referral for further intervention (re-casting or surgical review) was required.**  **Details include: Clinical examination composed observation, physical assessment and functional review; it included assessment of passive and active range of motion (plantiflexion, dorsiflexion, eversion, inversion of the foot and knee extension), muscle strength tests of the calf and evertors of the foot), heel raises, squatting ability and gait analysis (walking and running).** |
|  | 11 | Rationale for choosing the reference standard (if alternatives exist)  **The most frequently used approach to measuring whether the Ponseti method has been successful (or not) is clinical assessment. In sub-Saharan Africa 68% to 98% of cases are reported to have a successful outcome with the Ponseti method (4)** |
|  | 12a | Definition of and rationale for test positivity cut-offs or result categories of the index test, distinguishing pre-specified from exploratory  **References given and limitations outlined in the discussion section:**  **The threshold for diagnostic accuracy was set at 70% for the three scores with continuous scales (14) and positive/negative for the binary outcomes (7).** |
|  | 12b | Definition of and rationale for test positivity cut-offs or result categories of the reference standard, distinguishing pre-specified from exploratory  **The threshold for diagnostic accuracy was based on previous studies and was defined prior to the study. It was set at 70% for the three scores with continuous scales (14) and positive/negative for the binary outcomes (7)** |
|  | 13a | Whether clinical information and reference standard results were available to the performers or readers of the index test  **Categorisation of information for each test is provided in the web appendices** |
|  | 13b | Whether clinical information and index test results were available to the assessors of the reference standard  **The clinical information and test results were available to the assessors of the reference standard and the reference standard was consensus on a decision for further referral or not** |
| Analysis | 14 | Methods for estimating or comparing measures of diagnostic accuracy  **Sensitivity, specificity, positive and negative predictive values were calculated for the five measures and compared to full clinical assessment (gold standard). The results are categorised as true positive (TP), false positive (FP) (referred but not needed), true negative (TN), and false negative (FN)(should have been referred but missed).** |
|  | 15 | How indeterminate index test or reference standard results were handled  **Two assessments by individual physiotherapists and subsequent consensus was required for the standard reference result to avoid indeterminate results** |
|  | 16 | How missing data on the index test and reference standard were handled  **Missing data were excluded and noted on all tables of results** |
|  | 17 | Any analyses of variability in diagnostic accuracy, distinguishing pre-specified from exploratory  **Post hoc analyses was not performed** |
|  | 18 | Intended sample size and how it was determined  **Sample size was based on of the number of children first attending a single clinic over a period of 25 months and the follow up of these children. 31% (68/218) of the cohort attended for review and were assessed** |
| **Results** | | |
| Participants | 19 | Flow of participants, using a diagram  **Reference of flow diagram in original cohort analysis is given and explanation of sampling technique included “The phone numbers of all carers of the cohort children were extracted from the clinic records in January 2017 and contact with them was attempted at least three times. Caregivers and their children were invited to attend the study. The children were between 3.5 and 5 years from initial casting.”** |
|  | 20 | Baseline demographic and clinical characteristics of participants  **Included in the first paragraph of results** |
|  | 21a | Distribution of severity of disease in those with the target condition  **Included in the first paragraph of results** |
|  | 21b | Distribution of alternative diagnoses in those without the target condition  **n/a due to selection criteria** |
|  | 22 | Time interval and any clinical interventions between index test and reference standard  **Independent assessment occurred on the same day between the index test and the reference standard so as to avoid any change in condition of the clubfoot position** |
| Test results | 23 | Cross tabulation of the index test results (or their distribution) by the results of the reference standard  **Outlined in results and full tables included in web appendix** |
|  | 24 | Estimates of diagnostic accuracy and their precision (such as 95% CIs)  **Provided in results** |
|  | 25 | Any adverse events from performing the index test or the reference standard  **No adverse events occurred as a result of any of the outcome measures undertaken** |
| **Discussion** | | |
|  | 26 | Study limitations, including sources of potential bias, statistical uncertainty and generalisability  **Included in discussion** |
|  | 27 | Implications for practice, including the intended use and clinical role of the index test  **Included in discussion** |
| **Other information** | | |
|  | 28 | Registration number and name of registry  **This cohort study was not registered** |
|  | 29 | Where the full study protocol can be accessed  **The design and rationale of the study was not previously published** |
|  | 30 | Sources of funding and other support; role of funders  **Included in acknowledgements** |
