# Supplementary material for: A comparison of outcome measures used to report clubfoot treatment with the Ponseti method: results from a cohort in Harare, Zimbabwe
Source: BMC Musculoskelet Disord. 2018 Dec 22;19:450. doi: 10.1186/s12891-018-2365-3 (PMC6303847; doi:10.1186/s12891-018-2365-3)
Supplement: Supplementary file 2 — Summary of outcomes: Roye score. Individual category calculations for the Roye score. (DOCX 24 kb) [file 12891_2018_2365_MOESM2_ESM.docx]

**Additional File 2: Summary of outcomes: Roye Score**

The total score includes all 10 items. Scores are computed by reverse-coding, summing the responses, and then dividing the result by the number of items completed, ie, scores are a mean of the responses. For ease of interpretation, the scores are linearly transformed to a 0 to 100 scale with 100 being best, so that 1 = 0, 2 = 33.3, 3 = 66.7, and 4 = 100 for questions with four possible answers. The one yes-or-no item (ever complains of pain in affected foot) was coded yes = 0 or no = 100.

**Results of Roye score for cohort (N=66)**

| **Roye score (cohort followed up, n=66*)** | **Very satisfied N (%)** | **Somewhat satisfied  N (%)** | **Somewhat dissatisfied N (%)** | **Dissatisfied N (%)** | Satisfaction | **Individual Score Mean % (95%CI)** | **Subscale Mean % (95%CI)** |
| --- | --- | --- | --- | --- | --- | --- | --- |
| 1. satisfied with status of foot | 42 (64) | 15 (23) | 5 (7) | 4 (6) |  | 81  (74 - 89) | 85  (80 - 90) |
| 2. satisfied with appearance | 43 (65) | 14 (21) | 5 (8) | 4 (6) |  | 82  (75 - 89) |  |
|  | **Never N(%)** | **Sometimes N(%)** | **Usually N(%)** | **Always N(%)** |  |  |  |
| 3. How often is your child teased? | 54 (82) | 8 (12) | 4 (6) | 0 (0) |  | 91  (87 - 96) |  |
| 4. How often does your child have problems finding shoes that fit? | 49 (74) | 10 (15) | 3 (5) | 4 (6) |  | 86  (79 - 93) |  |
| 5. How often does your child have problems finding shoes that he or she likes? | 45 (68) | 15 (22) | 3 (5) | 3 (5) |  | 85  (78 - 91) |  |
|  | **No N (%)** | **Yes**  **N(%)** |  | |  |  |  |
| 6. Does your child ever complain of pain in his or her [affected] foot? | 26 (39) | 40 (61) |  |  | Function | 61  (49 - 73) | 87  (82 - 91) |
|  | **Not at all limited N(%)** | **Somewhat limited N(%)** | **Moderately limited N(%)** | **Very limited N(%)** |  |  |  |
| 7. How limited is your child in his or her ability to walk? | 60 (91) | 5 (8) | 1 (1) | 0 (0) |  | 96  (94 - 99) |  |
| 8. How limited is your child in his or her ability to run? | 52 (79) | 10 (15) | 3 (5) | 1 (1) |  | 90  (84 - 96) |  |
|  | **Never N(%)** | **Sometimes N(%)** | **Usually  N(%)** | **Always N(%)** |  |  |  |
| 9. How often does your child complain of pain during heavy exercise? | 46 (70) | 18 (27) | 1 (1.5) | 1 (1.5) |  | 88  (84 - 93) |  |
| 10. How often does your child complain of pain during moderate exercise? | 61 (92.5) | 4 (6) | 1 (1.5) | 0 (0) |  | 97  (94 - 100) |  |

*results from 2 children missing as attended without primary carer

**Results of Roye score for those who completed casting (N=61)**

| **Roye score (completed casting) n=59** | **Very satisfied N (%)** | **Somewhat satisfied  N (%)** | **Somewhat dissatisfied N (%)** | **Dissatisfied N (%)** | Satisfaction | **Individual Mean % (95%CI)** | **Subscale Mean % (95%CI)** |
| --- | --- | --- | --- | --- | --- | --- | --- |
| 1. satisfied with status of foot | 41 (67.2) | 13 (21.3) | 5 (8.2) | 2 (3.3) |  | 84  (77 - 91) | 87  (83 - 92) |
| 2. satisfied with appearance | 42 (68.8) | 12 (19.7) | 5 (8.2) | 2 (3.3) |  | 85  (78 - 91) |  |
|  | **Never N(%)** | **Sometimes N(%)** | **Usually N(%)** | **Always N(%)** |  |  |  |
| 3. How often is your child teased? | 52 (85.2) | 7 (11.5) | 2 (3.3) | 0 (0) |  | 94  (90 - 98) |  |
| 4. How often does your child have problems finding shoes that fit? | 48 (78.7) | 8 (13.1) | 2 (3.3) | 3 (4.9) |  | 89  (82 - 95) |  |
| 5. How often does your child have problems finding shoes that he or she likes? | 42 (68.9) | 14 (22.9) | 3 (4.9) | 2 (3.3) |  | 86  (79 - 92) |  |
|  | **No N (%)** | **Yes N (%)** |  | |  |  |  |
| 6. Does your child ever complain of pain in his or her [affected] foot? | 39 (63.9) | 22 (36.1) |  |  | Function | 64  (52 - 76) | 88  (84 - 92) |
|  | **Not at all limited N (%)** | **Somewhat limited N (%)** | **Moderately limited N (%)** | **Very limited**  **N (%)** |  |  |  |
| 7. How limited is your child in his or her ability to walk? | 56 (91.8) | 5 (8.2) | 0 (0) | 0 (0) |  | 97  (95 - 100) |  |
| 8. How limited is your child in his or her ability to run? | 49 (80.3) | 10 (16.5) | 1 (1.6) | 1 (1.6) |  | 92  (87 - 97) |  |
|  | **Never N (%)** | **Sometimes N (%)** | **Usually  N (%)** | **Always N (%)** |  |  |  |
| 9. How often does your child complain of pain during heavy exercise? | 44 (72.1) | 16 (26.2) | 0 (0) | 1 (1.7) |  | 90  (85 - 94) |  |
| 10. How often does your child complain of pain during moderate exercise? | 58 (95.1) | 3 (4.9) | 0 (0) | 0 (0) |  | 98  (97 - 100) |  |
|  |  |  |  |  |  |  |  |

**Results of Roye score for those who completed ≥2 years bracing (N=38)**

| **Roye score (completed ≥2 years bracing) n=36** | **Very satisfied N (%)** | **Somewhat satisfied  N (%)** | **Somewhat dissatisfied N (%)** | **Dissatisfied N (%)** | Satisfaction | **Individual Mean % (95%CI)** | **Subscale Mean % (95%CI)** |
| --- | --- | --- | --- | --- | --- | --- | --- |
| 1. satisfied with status of foot | 27 (75%) | 7 (19%) | 1 (3%) | 1 (3%) |  | 89  (81 -97) | 89  (82 – 95) |
| 2. satisfied with appearance | 28 (78%) | 6 (16%) | 1 (3%) | 1 (3%) |  | 90  (82 – 97) |  |
|  | **Never N(%)** | **Sometimes N(%)** | **Usually N(%)** | **Always N(%)** |  |  |  |
| 3. How often is your child teased? | 32 (89%) | 3 (8%) | 1 (3%) | 0 (0%) |  | 95  (91 – 100) |  |
| 4. How often does your child have problems finding shoes that fit? | 27 (75%) | 5 (14%) | 1 (3%) | 3 (8%) |  | 85  (75 – 95) |  |
| 5. How often does your child have problems finding shoes that he or she likes? | 25 (69%) | 7 (19%) | 2 (6%) | 2 (6%) |  | 84  (75 – 94) |  |
|  | **No N (%)** | **Yes N (%)** |  | |  |  |  |
| 6. Does your child ever complain of pain in his or her [affected] foot? | 27 (75%) | 9 (25%) |  |  | Function | 75  (60 – 90) | 91  (87 – 96) |
|  | **Not at all limited N (%)** | **Somewhat limited**  **N (%)** | **Moderately limited N (%)** | **Very limited**  **N (%)** |  |  |  |
| 7. How limited is your child in his or her ability to walk? | 34 (94%) | 2 (6%) | 0 (0%) | 0 (0%) |  | 98  (96 – 100) |  |
| 8. How limited is your child in his or her ability to run? | 29 (81%) | 6 (17%) | 0 (0%) | 1 (3%) |  | 92  (84 – 98) |  |
|  | **Never N (%)** | **Sometimes N (%)** | **Usually  N (%)** | **Always N (%)** |  |  |  |
| 9. How often does your child complain of pain during heavy exercise? | 28 (78%) | 8 (22%) | 0 (0%) | 0 (0%) |  | 93  (88 – 97) |  |
| 10. How often does your child complain of pain during moderate exercise? | 34 (94%) | 2 (6%) | 0 (0%) | 0 (0%) |  | 98  (96 – 100) |  |

*data from 2 children missing
