# Supplementary material for: A comparison of outcome measures used to report clubfoot treatment with the Ponseti method: results from a cohort in Harare, Zimbabwe
Source: BMC Musculoskelet Disord. 2018 Dec 22;19:450. doi: 10.1186/s12891-018-2365-3 (PMC6303847; doi:10.1186/s12891-018-2365-3)
Supplement: Supplementary file 3 — Summary of outcomes: Bangla score. Individual category calculations for the Bangla score. (DOCX 23 kb) [file 12891_2018_2365_MOESM3_ESM.docx]

**Additional File 3: Summary of outcomes: Bangla score**

‘Grading’ Very good Good Fair Poor

% 85-100 70 - 85 60-70 <50

1. Parent rating subscore /5 where yes = +1, don’t know = 0 and no = -1
2. Gait assessment subscore /4 where yes = +1, not fully – 0, no = -1
3. Clinical examination /2 where valgus/>DF = +1, straight/90 degrees = 0 and varus/<0 df = -1

Scores for bilateral cases were halved to achieve same scale/foot for section C/clinical examination

Total score converted to % indicated quality/grade of individual child’s results from treatment

**Results of Bangla score for entire cohort (n=68)**

| A. Parent rating | Yes +1 N (%) | Don't know 0 N (%) | No -1 N (%) | Mean scores (%) | Rating Number |
| --- | --- | --- | --- | --- | --- |
| 1. Happy with child’s feet | 57 (84) | 2 (3) | 9 (13) | 71 | "Good" |
| 2. Recommend to others? | 68 (100) | 0 (0) | 0 (0) | 100 |  |
| 3. Does child play with others? | 68 (100) | 0 (0) | 0 (0) | 100 |  |
| 4. Does child wear shoes of choice? | 53 (78) | 0 (0) | 15 (22) | 56 |  |
|  | Yes -1 N (%) | Don't know 0 N (%) | No +1 N (%) |  |  |
| 5. Does child have pain? | 22 (32) | 0 (0) | 46 (68) | 35 |  |
| **Parental Rating sub score** | | | | 72% |  |
| B. Gait assessment | Yes +1 N (%) | Not fully/ with assistance 0 N (%) | No -1 N (%) | Mean scores (%) | "Very good" |
| 6. Squatting | 66 (97) | 0 (0) | 2 (3) | 94 |  |
| 7. Walking | 67 (99) | 1 (1) | 0 (0) | 99 |  |
| 8. Running | 67 (99) | 0 (0) | 1 (1) | 98 |  |
| 9. Up/down steps | 67 (99) | 0 (0) | 1 (1) | 98 |  |
| **Gait assessment sub score** | | | | 97% |  |
| C. Clinical examination | Valgus +1 N (%) | Straight 0 N (%) | Varus -1 N (%) | Mean scores (%) | “Fair” |
| 10. Heel position - left * n=53 | 2 (4) | 32 (60) | 19 (36) | 32 |  |
| Heel position - right ** n=48 | 4 (8) | 28 (58) | 16 (34) | 25 |  |
|  | >0 dorsiflexion +1 N (%) | 0/90 degrees 0 N (%) | <0 dorsiflexion N (%) | Mean scores (%) |  |
| 11. Ankle range - left* n=53 | 28 (53) | 17 (32) | 8 (15) | 38 |  |
| Ankle range - right** n=48 | 23 (48) | 15 (31) | 10 (21) | 27 |  |
| **Clinical examination sub score (35 bilateral)** | | | | 61% |  |
| **Total score** | | | | **77%** | “Good” |

**Bangla score results from children who completed casting (n=61)**

| A. Parent rating | Yes +1 N (%) | Don't know 0 N (%) | No -1 N (%) | Mean scores (%) | Rating Number |
| --- | --- | --- | --- | --- | --- |
| 1. Happy with child’s feet | 52 (85) | 2 (3) | 7 (12) | 75 | "Very good" |
| 2. Recommend to others? | 61 (100) | 0 (0) | 0 (0) | 100 |  |
| 3. Does child play with others? | 61 (100) | 0 (0) | 0 (0) | 100 |  |
| 4. Does child wear shoes of choice? | 49 (80) | 0 (0) | 12 (20) | 59 |  |
|  | Yes -1 N (%) | Don't know 0 N (%) | No +1 N (%) |  |  |
| 5. Does child have pain? | 18 (30) | 0 (0) | 43 (70) | 40 |  |
| **Parental Rating sub score** | | | | 75% |  |
| B. Gait assessment | Yes +1 N (%) | Not fully/ with assistance 0 N (%) | No -1 N (%) | Mean scores (%) | "Very good" |
| 6. Squatting | 60 (98) | 1 (2) | 0 (0) | 97 |  |
| 7. Walking | 61 (100) | 0 (0) | 0 (0) | 100 |  |
| 8. Running | 61 (100) | 0 (0) | 0 (0) | 100 |  |
| 9. Up/down steps | 61 (100) | 0 (0) | 0 (0) | 100 |  |
| **Gait assessment sub score** | | | | 99% |  |
| C. Clinical examination | Valgus +1 N (%) | Straight 0 N (%) | Varus -1 N (%) | Mean scores (%) | “Very good” |
| 10. Heel position - left * n=46 | 2 (4) | 28 (61) | 16 (35) | 38 |  |
| Heel position - right **n=42 | 3 (7) | 25 (60) | 14 (33) | 41 |  |
|  | >0 dorsiflexion +1 N (%) | 0/90 degrees 0 N (%) | <0 dorsiflexion N (%) | Mean scores (%) |  |
| 11. Ankle range - left* n=46 | 25 (54) | 16 (35) | 5 (11) | 42 |  |
| Ankle range - right** n=42 | 21 (50) | 14 (33) | 7 (17) | 33 |  |
| **Clinical examination sub score (30 bilateral)** | | | | 77% |  |
| **Total score** | | | | **84%** | “Very good” |

**Bangla score results from children who completed ≥2 years bracing (n=38)**

| A. Parent rating | Yes +1 N (%) | Don't know 0 N (%) | No -1 N (%) | Mean scores (%) | Rating Number |
| --- | --- | --- | --- | --- | --- |
| 1. Happy with child’s feet | 34 (89%) | 0 (0%) | 4 (11%) | 79 | “Very good” |
| 2. Recommend to others? | 38 (100%) | 0 (0%) | 0 (0%) | 100 |  |
| 3. Does child play with others? | 38 (100%) | 0 (0%) | 0 (0%) | 100 |  |
| 4. Does child wear shoes of choice? | 31 (82%) | 0 (0%) | 7 (18%) | 63 |  |
|  | Yes -1 N (%) | Don't know 0 N (%) |  |  |  |
| 5. Does child have pain? | 28 (74%) | 0 (0%) | 10 (26%) | 47 |  |
| **Parental Rating sub score** | | | | **78** |  |
| B. Gait assessment | Yes +1 N (%) | Not fully/ with assistance 0 N (%) | No -1 N (%) | Mean scores (%) | “Very good” |
| 6. Squatting | 38 (100%) | 0 (0%) | 0 (0%) | 100 |  |
| 7. Walking | 38 (100%) | 0 (0%) | 0 (0%) | 100 |  |
| 8. Running | 38 (100%) | 0 (0%) | 0 (0%) | 100 |  |
| 9. Up/down steps | 38 (100%) | 0 (0%) | 0 (0%) | 100 |  |
| **Gait assessment sub score** | | | | 100 |  |
| C. Clinical examination | Valgus +1 N (%) | Straight 0 N (%) | Varus -1 N (%) | Mean scores (%) |  |
| 10. Heel position - left * n=46 | 1 (4%) | 22 (73%) | 7 (23%) | 20 |  |
| Heel position - right **n=42 | 1 (4%) | 17 (68%) | 7 (28%) | 24 |  |
|  | >0 dorsiflexion +1 N (%) | 0/90 degrees 0 N (%) | <0 dorsiflexion N (%) | Mean scores (%) |  |
| 11. Ankle range - left* n=46 | 19 (63%) | 10 (33%) | 1 (4%) | 60 |  |
| Ankle range - right** n=42 | 15 (60%) | 10 (40%) | 0 (0%) | 60 |  |
| **Clinical examination sub score (30 bilateral)** | | | | 82 |  |
| **Total score** | | | | **76** | “Very good” |
