# Supplementary material for: A comparison of outcome measures used to report clubfoot treatment with the Ponseti method: results from a cohort in Harare, Zimbabwe
Source: BMC Musculoskelet Disord. 2018 Dec 22;19:450. doi: 10.1186/s12891-018-2365-3 (PMC6303847; doi:10.1186/s12891-018-2365-3)
Supplement: Supplementary file 4 — Summary of outcomes: ACT score. .Individual category calculations for the ACT score. (DOCX 16 kb) [file 12891_2018_2365_MOESM4_ESM.docx]

**Additional File 4: ACT score**

| **Score** | **The foot is plantigrade** | **Does your child complain of pain in their affected foot?** | **Can your child wear shoes of your/their choice?** | **How satisfied are you with your child’s foot?** |
| --- | --- | --- | --- | --- |
| **3** | **More than plantigrade ie some dorsiflexion** | **No** | **Always** | **Very satisfied** |
| **2** | **Plantigrade** | **Yes but it does not limit their activity** | **Usually** | **Somewhat satisfied** |
| **1** | **Does not reach plantigrade** | **Yes and it sometimes limits their activity** | **Sometimes** | **Somewhat dissatisfied** |
| **0** | **Less than plantigrade with adduction, cavus or varus** | **Yes and it often limits their activity** | **Never** | **Very dissatisfied** |

**ACT score distribution**

|  | | 3 | 2 | 1 | 0 |
| --- | --- | --- | --- | --- | --- |
|  |  | Children  N (%) | Children  N (%) | Children  N (%) | Children  N (%) |
| Cohort followed up (n=68) | Foot is plantigrade | 33 (49%) | 22 (32%) | 7 (10%) | 6 (9%) |
|  | Complain of pain | 44 (65%) | 18 (27%) | 5 (7%) | 1 (1%) |
|  | Wears shoe of choice | 47 (69%) | 13 (19%) | 6 (9%) | 2 (3%) |
|  | Satisfied with foot | 42 (62%) | 14 (21%) | 9 (13%) | 3 (4%) |
| Completed casting (n=63) | Foot is plantigrade | 31 (49%) | 22 (35%) | 7 (11%) | 3 (5%) |
|  | Complain of pain | 43 (68%) | 15 (24%) | 5 (8%) | 0 (0%) |
|  | Wears shoe of choice | 45 (71%) | 12 (19%) | 6 (10%) | 0 (0%) |
|  | Satisfied with foot | 41 (65%) | 12 (19%) | 9 (14%) | 1 (2%) |
| Completed ≥2 years bracing (n=38) | Foot is plantigrade | 22 (58%) | 15 (39%) | 1 (3%) | 0 (0%) |
|  | Complain of pain | 27 (71%) | 9 (24%) | 2 (5%) | 0 (0%) |
|  | Wears shoe of choice | 27 (71%) | 8 (21%) | 3 (8%) | 0 (0%) |
|  | Satisfied with foot | 28 (74%) | 6 (16%) | 4 (10%) | 0 (0%) |

**Interpretation of ACT score in similar manner to Roye score - the scores are linearly transformed to a 0 to 100 scale with 100 being best, so that 1 = 0, 2 = 33.3, 3 = 66.7, and 4 = 100
