# Supplementary material for: A comparison of outcome measures used to report clubfoot treatment with the Ponseti method: results from a cohort in Harare, Zimbabwe
Source: BMC Musculoskelet Disord. 2018 Dec 22;19:450. doi: 10.1186/s12891-018-2365-3 (PMC6303847; doi:10.1186/s12891-018-2365-3)
Supplement: Supplementary file 5 — Summary of outcomes: Relapse score. Individual category calculations for the Relapse score. (DOCX 15 kb) [file 12891_2018_2365_MOESM5_ESM.docx]

**Additional File 5: Relapse score**

**Assessment of relapse**

| Relapse type | Cohort Children (n=68) N (%) | Children completed casting (n=63) N (%) | Children completed ≥2years bracing (n=32)* N (%) |
| --- | --- | --- | --- |
| No relapse | 37 (55%) | 36 (57%) | 18 (56%) |
| 1A (decrease in ankle dorsiflexion from 15degrees to neutral with knee extension) | 13 (19%) | 13 (21%) | 6 (19%) |
| 2A Dynamic forefoot adduction or supination of foot | 1 (1%) | 1 (2%) | 0 (0%) |
| 1B Fixed equinus of any degree (passive correction to neutral not possible) | 5 (7%) | 4 (6%) | 4 (13%) |
| 2B Fixed adduction of forefoot and midfoot (fixed lateral curvature) | 0 (0%) | 0 (0%) | 0 (0%) |
| 3 (Two or more fixed deformities) | 12 (18%) | 9 (14%) | 4 (13%) |
| *missing data from 6 children |  |  |  |
